# Rectal Hyposensitivity Is Associated With a Defecatory Disorder But Not Delayed Colon Transit Time in a Functional Constipation Population

**DOI:** 10.1097/MD.0000000000003667

**Published:** 2016-05-13

**Authors:** Ting Yu, Dong Qian, Yongping Zheng, Ya Jiang, Ping Wu, Lin Lin

**Affiliations:** From the Department of Gastroenterology (TY, YZ, YJ, PW, LL); Pancreas Center (DQ), the First Affiliated Hospital of Nanjing Medical University; Pancreas Institute (DQ), Nanjing Medical University; and Qinglongshan Mental Hospital (PW), Nanjing, China.

## Abstract

The physiological mechanism of functional constipation (FC) includes defecatory disorders and delayed colon transit. About 18% to 68% constipated patients may have rectal hyposensitivity (RH). We performed this study to investigate the association between RH and functional defecatory disorder (FDD) as well as that between RH and delayed colon transit in FC patients.

A total of 218 FC patients were enrolled. The constipation severity instrument (CSI) was used to assess constipation symptoms. High-resolution anorectal manometry (HR-ARM), defecography, balloon expulsion tests, and colon transit studies were performed for each patient. RH was defined as 1 or more sensory threshold pressures raised beyond the normal range based on HR-ARM. We investigated the association between RH and constipation symptoms, and the occurrence of FDD and delayed CTT. Ninety FDD patients completed the initial phase of biofeedback treatment (BFT). We investigated the association between RH and the effect of BFT.

Totally 122 (56.0%) patients had RH. The total CSI (49.82 ± 1.09 vs 41.25 ± 1.55, *P* = 0.023) and obstructive defecation subscale scores (23.19 ± 0.69 vs 17.07 ± 0.90, *P* < 0.001) were significantly higher in RH than in non-RH patients. No significant difference was observed in slow transit symptoms (21.77 ± 0.72 vs 19.90 ± 0.85, *P* = 0.121) or abdominal pain (6.85 ± 2.61 vs 5.00 ± 1.04, *P* = 0.380). The frequency of prolonged CTT was not significantly different between RH and non-RH groups (54.1% vs 58.3%, *P* = 0.403). RH patients rated more occurrence of FDD (72.1% vs 53.1%, *P* = 0.014) and dysynergic defecation (79.8% vs 50.2%, *P* = 0.004) than non-RH patients, whereas no differences were seen for inadequate defecatory propulsion (59.2% vs 55.0%, *P* = 0.589). After BFT, the proportion of “no effect” was significantly higher in the RH group than in the non-RH group (22.4% vs 9.4%, *P* = 0.010).

RH is associated with obstructive defecation symptoms and the occurrence of FDD. Further studies are needed to detect the mechanism of RH's effect on BFT and FC.

## INTRODUCTION

Functional constipation (FC) is a common gastrointestinal disorder. In Western countries, the prevalence of FC was reported as between 2% and 28%.^1–3^ The overall prevalence among Chinese adults of all ages is 16% to 20%.^4^ About 50% of FC patients complain of difficulty with defecation^5^ and may have a functional defecatory disorder (FDD).^6^ These patients have obstructive defecation symptoms, such as severe straining, sensation of a “blockage,” or incompletion on attempted defecation. The physiological mechanisms of FDD include paradoxical contraction or inadequate relaxation of the pelvic muscles (dysynergic defecation) and inadequate rectum propulsion during attempted defecation.^7^ Another important cause of FC is disordered colonic motor function.^8^ In addition to prolonged colon transit time, patients typically experience abdominal distension, bloating, and discomfort, as well as a lack of the urge to defecate.^9^

Rectal hyposensitivity (RH) refers to elevation of sensory thresholds beyond the normal range, resulting in rectal sensory dysfunction.^10^ About 18% to 68% constipated patients may have RH.^11^ RH has been assumed to be an important cause of FDD and may predict poor outcome of biofeedback therapy (BFT) for FDD.^10,12–14^ However, the results from 2 recent large clinical studies did not support this conclusion.^15,16^ Lee et al^15^ and Wijffels et al^16^ reported that RH was not associated with the incidence of obstructed defecation in constipated patients. It is worth noting that a number of patients included in their studies were characterized by internal rectal prolapse, rectal intussusceptions, or rectocele. For these patients, anatomical abnormalities may be more important. Besides, RH may lead to the development of constipation by influencing colonic motility. Delayed colonic transit is found in up to one-third of patients with RH.^17^

Based on these conflicting results, we designed the present study to determine the impact of RH in a Chinese FC population.

## METHODS

### Participants

A total of 240 patients with constipation, who attended the Department of Gastroenterology at the First Affiliated Hospital of Nanjing Medical University between January 2012 and December 2014, were recruited for the study. The inclusion criteria were: (1) FC diagnosed using the Rome III criteria in the preceding 3 months, whose onset was at least 6 months before diagnosis (without the use of laxatives) of ≥ 2 of the following complaints: passing a stool ≤ 3 times a week, straining during defecation, feeling of incomplete evacuation, hard stools, feeling of anorectal obstruction, manual maneuvers require to expedite defecation on at least 25% of occasions; (2) patients should be aged between 18 and 75 years; (3) patients should have no history of abdominal and anorectal surgery; (4) no abnormality at endoscopy or radiographic examination of the gastrointestinal tract; (5) normal laboratory routine tests; (6) absence of systemic disease; (7) no history of psychotropic disease; (8) no evidence of internal rectal prolapse or rectal intussusceptions or rectocele at defecography; (9) completed constipation severity instrument questionnaire (CSI), high-resolution anorectal manometry (HR-ARM), balloon expulsion test (BET), and colon transit time (CTT). Totally, 56 volunteers were withdrawn from the study because they did not fulfill the inclusion criteria. Finally, 218 patients were included (137 [63%] female, 81 [37%] male).

FC patients with defecation disorders were suggested to receive biofeedback therapy (BFT) in our hospital. Finally, 90 patients completed the first phase of treatment (10 sessions) (53 [59%] female, 37 [41] male).

Sixty age- and gender-matched volunteers were recruited from January to December 2014. Inclusion criteria were as follows: (1) normal defecation within 6 months before enrollment (stool frequency <3 times/day and >3 times/week, defecation time <10 minutes, no straining during defecation, no sensation of incomplete evacuation, no anorectal obstruction, no manual maneuvers to facilitate defecation, abdominal pain with defecation, no fecal incontinence; (2) to (7) were the same as the inclusion criteria for FC patients; (8) completed HR-ARM to detect rectal sensation. Finally, 54 volunteers were included (35 [65%] female, 19 [35] male).

The cross-sectional study was approved by the Ethics Committee of the First Affiliated Hospital of Nanjing Medical University (2016-SRFA-064). In addition, all identifying information about the patients was removed from our records before the analyses, to protect patient privacy. This study was conducted according to the principles expressed in the Declaration of Helsinki.

## MEASURES

### Constipation Severity Instrument

A self-administered modified CSI was used to assess constipation symptoms.^18–20^ The modified CSI, which is a reliable and valid instrument to evaluate constipation, includes 3 subscales: obstructive defecation (6 scores), colonic inertia (6 scores), and pain (4 scores). The scores range for each subscale is 0 to 28, 0 to 29, and 0 to16 (overall CSI score range, 0 to74 points), respectively. The higher the total CSI score, the more severe the constipation symptoms.

### Defecography

Barium paste (∼150 cc) was inserted into the rectum. Images of the anorectal segment were obtained at rest, during coughing and during attempts to bear down as if to defecate using lateral video fluoroscopy.^7^ A retention of ≥50% contrast with or without a poor rectal stripping wave defined an abnormal defecography.

### Rectal Balloon Expulsion Test

We measured the time taken for patients to expel from their rectum a balloon filled with 50 cc of warm water while seated a commode in privacy. If the subject could not expel the balloon in 1 minute, the balloon was removed.^21,22^

### Colonic Transit Study

Colonic transit was assessed using radiopaque marker techniques. In brief, patients ingested a single capsule containing 24 radiopaque markers (tube-shaped, with a diameter of 2 mm and a length of 6 mm) on day 1, and a supine x-ray of the abdomen was obtained on day 3 (72 hours later). The x-rays were analyzed to assess the number and distribution of the markers. Patients were deemed positive for evidence of delayed colon transit when there were >4 markers at 72 hours throughout the colon.^23,24^

### High-Resolution Anorectal Manometry

A novel solid-state HR-ARM device (Manoscan AR 360; Given Imaging, Yoquem, Israel) with 12 sensors was used. Patients were studied in the left lateral decubitus position, with hips flexed to 90°, after defecation. The catheter was placed with the rectal balloon 3 cm proximal to the superior aspect of the anal sphincter. Parameters were assessed in the following order: anal and rectal pressure at rest (20–30 seconds), during squeeze (3 attempts for a maximum duration of 20–30 seconds), bearing down as in defecation (typically 20–30 seconds, 3 times).^25^

We evaluated rectal sensation by progressively distending the rectal balloon from 0 to 50 mL simultaneously, and recorded threshold volumes for first sensation, urgency, and maximum discomfort. Normal values for these 3 parameters in females and males referred to the upper limit of 95% of the reference range of healthy volunteers.

### Functional Defecation Disorder Definition

FDD was defined according to the Rome III criteria (diagnostic criteria for FC and at least 2 of the following abnormalities: (1) evidence of impaired evacuation, based on BET or defecography; (2) inappropriate contraction or failure to relax the pelvic floor muscles during repeated attempts at defecation (dysynergic defecation), as measured by HR-ARM or defecography; (3) inadequate propulsive forces (inadequate defecatory propulsion), as assessed by HR-ARM or defecography).^6^

### Biofeedback Training

Patients accepted BFT using the Orion Platinum biofeedback equipment (SRS Medical Systems Inc., Redmond, WA), according to the instructions from a therapist. Patients received 10 sessions of 1-h BFT was provided to the patients: once every other day in the first 2 weeks, and then 2 to 3 times every week.^26,27^ The final analysis only included patients that completed 10 sessions of training and did not take any laxatives during the training process. At completion of the BFT session, the clinical efficacy was assessed. To obtain a valid score, the decreasing index between the pretraining and post-training CSI scores was divided by the pretraining score. Clinical efficacy was considered “very efficacious” when the score was >0.50, and “efficacious” when the score was between 0.25 and 0.50. “No efficacy” corresponded to a score of <0.25.

### Statistical Analysis

Differences in age and rectal sensation metrics between healthy volunteers and FC patients, age, gender, CSI score, the occurrences of slow transit, FDD, dysynergic defecation, inadequate defecatory propulsion, and effectiveness of BFT between RH and non-RH groups were analyzed. All data were analyzed using SPSS Version 20.0 (SPSS, Chicago, IL). Continuous variables are given as the mean ± standard error of the mean (SEM) and categorical variables as relative frequencies. Student's *t* test or the Mann–Whitney *U* test were used to compare continuous variables, and chi-squared test or Fisher's exact test for categorical variables. A *P* value <0.05 was considered statistically significant.

## RESULTS

### Comparison Between Healthy Volunteers and FC Patients

There were no significant differences between the 2 groups regarding age (53.7 ± 1.5 vs 51.7 ± 2.2 years, *P* = 0.874) and gender (*P* = 0.210). The disease duration of FC patients was 5.2 ± 1.0 years. A total of 150 patients had lost awareness of defecation and had no spontaneous bowel movements within the most recent 2 years. Also, 178 patients had a history of long-term use of stimulant laxatives. No patients had a history of anorectal surgery. The comparisons of rectal sensation metrics between the 2 groups are shown in Table 1. Threshold volumes for first sensation, urgency, and maximum discomfort were all significantly higher in FC patients compared with healthy volunteers (48.2 ± 2.6 vs 42.0 ± 3.6 cc, *P* = 0.013, 111.2 ± 5.9 vs 90.0 ± 2.3 cc, *P* < 0.001, 177.5 ± 6.1 vs 140.5 ± 5.1 cc, *P* < 0.001). The upper limit of 95% of the reference range for these 3 parameters of healthy volunteers is listed in Table 2. According to this, 82 (38%) FC patients had normal sensation, 122 (56%) had RH, and 14 (6%) had rectal hypersensitivity. We considered 96 (44%) patients as non-RH.

**TABLE 1:**
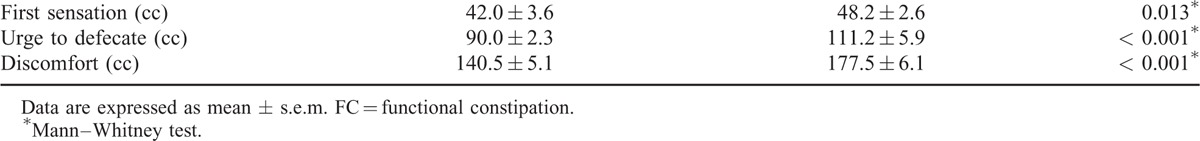
Comparison of Rectal Sensation Metrics Between Healthy Volunteers and FC Patients

**TABLE 2:**
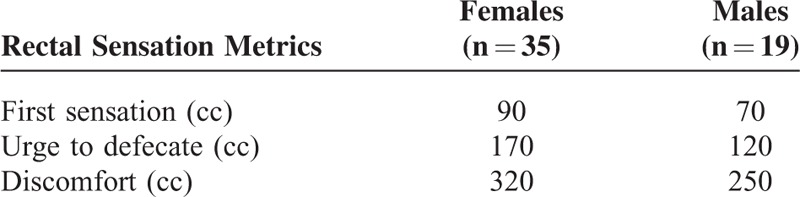
Upper Limit of 95% Reference of the Range for Rectal Sensation Metrics of Healthy Volunteers

## COMPARISON BETWEEN RH AND NON-RH PATIENTS

### Demographics

The RH and non-RH patients were similar in gender (72 [59.0%] females vs 60 [62.5%] females, *P* = 0.780). The RH patients were significantly older than the non-RH patients (55.3 ± 3.7 vs 46.7 ± 1.9, *P* = 0.001).

### CSI Scores

**FIGURE 1:**
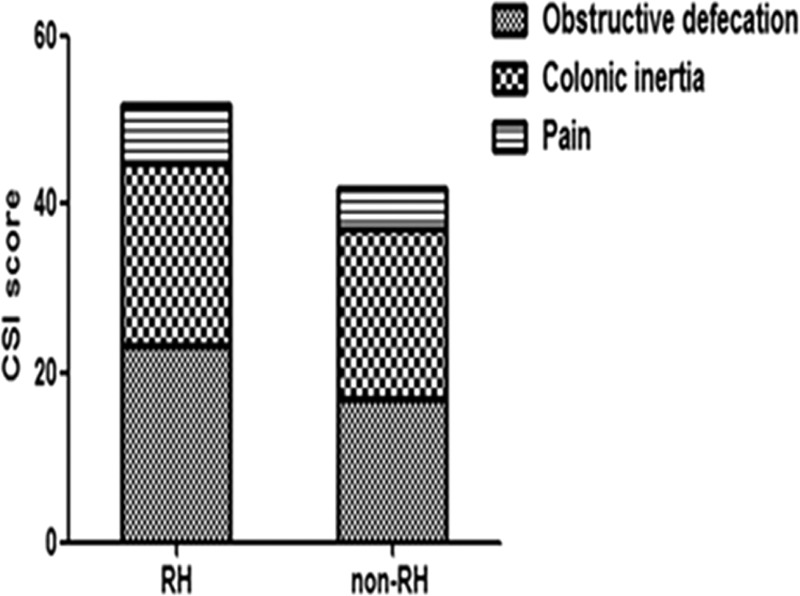
Comparison of CSI scores between RH and non-RH patients. Obstructive defecation scores were significantly different between the 2 groups (*P* < 0.001). Colonic inertia and pain scores were not significantly different (*P* = 0.121, *P* = 0.380). Total CSI scores were significantly different (*P* = 0.023). CSI = constipation severity instrument; RH = rectal hyposensitivity.

Differences in CSI scores between RH and non-RH patients are shown in Figure 1. We found that RH patients had more severe obstructive symptoms during defecation than non-RH patients (23.19 ± 0.69 vs 17.07 ± 0.90, *P* < 0.0001) and an overall higher total constipation symptom severity (49.82 ± 1.09 vs 41.25 ± 1.55, *P* = 0.023). However, there were no differences in slow transit symptoms (21.77 ± 0.72 vs 19.90 ± 0.85, *P* = 0.121) or abdominal pain (6.85 ± 2.61 vs 5.00 ± 1.04, *P* = 0.380).

### CTT

Overall, 122 patients (61.7%) presented with prolonged CTT, 66 (54.1%) in the RH group and 56 (58.3%) in the non-RH group. The frequency of prolonged CTT was not significantly different between the RH and non-RH groups (*P* = 0.403), as shown in Table 3.

**TABLE 3:**
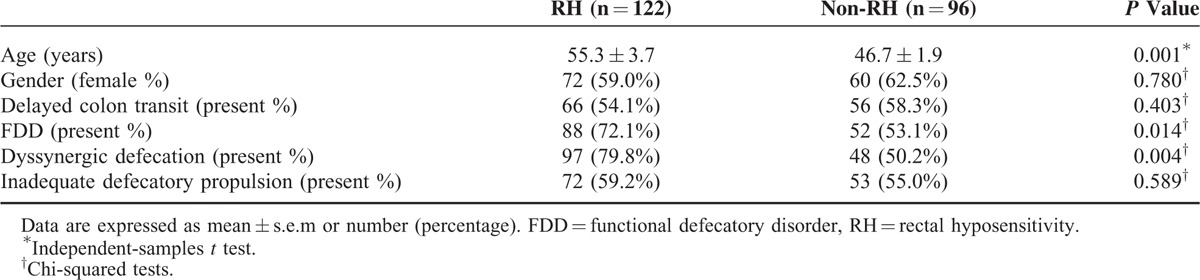
Comparison of the Demographics and Anorectal Physiology Between RH and non-RH Patients

### Occurrence of FDD

To detect FDD in FC patients, defecography, BET, and HR-ARM were used. Overall, 140 (64.2%) of all patients had FDD. Among the FDD patients, 145 (66.5%) patients presented with dysynergic defecation and 125 (57.3%) presented with inadequate defecatory propulsion. As shown in Table 3, RH patients showed a higher occurrence of FDD (*P* = 0.014) and dysynergic defecation (*P* = 0.004) than non-RH patients, whereas no differences between RH and non-RH patients were seen for inadequate defecatory propulsion (*P* = 0.589).

### Effect of BFT

Of the 140 FDD patients, 90 subjects completed the initial phase of treatment (10 sessions). There were 58 patients in the RH and 32 patients in the non-RH group. The mean age (50.32 ± 2.72 vs 52.94 ± 3.11, *P* = 0.530) and the proportion of females (34 females vs 19 females, *P* = 0.967) were not significantly different. We found that RH patients had more severe obstructive symptoms (28.12 ± 0.96 vs 23.27 ± 1.35, *P* = 0.013) and total constipation symptom severity (45.25 ± 1.52 vs 38.96 ± 2.22, *P* = 0.026) during defecation than non-RH patients. There were no differences in slow transit symptoms (14.57 ± 1.00 vs 13.84 ± 1.25, *P* = 0.946) or abdominal pain (3.68 ± 0.62 vs 3.97 ± 0.91, *P* = 0.186).

The results of the clinical efficacy after BFT of both groups are shown in Table 4. The proportion showing “no effect” was significant higher in the RH group than in the non-RH group (13 [22.4%] *vs* 3 [9.4%], *P* < 0.001).

**TABLE 4:**
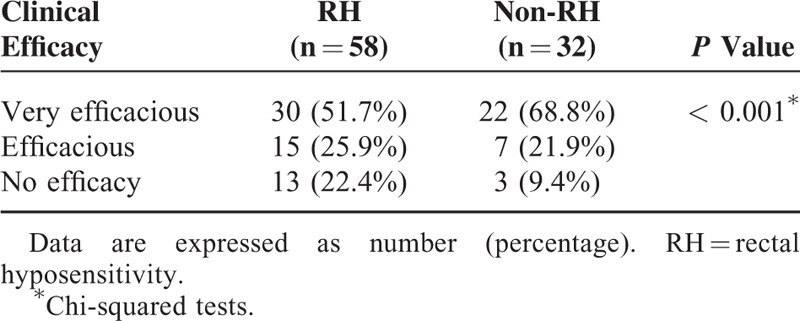
Comparison of BFT Efficacy Between RH and Non-RH Patients

## DISCUSSION

RH is presented as a diminished perception of rectal distension and defined as elevation of 1 or more of the 3 sensory thresholds (threshold volumes for first sensation, urgency, and maximum discomfort) in most studies.^15,16^ Our data suggested that all 3 sensory thresholds were significant higher in FC patients compared with healthy volunteers. According to the normative data derived from 60 healthy volunteers, we detected RH in 122/218 (56.0%) of patients with FC. These findings were consistent with previous studies, in which RH was reported in 18% to 68% of patients with constipation.^11–13^

Our result showed that the median age was higher in the RH group. This result suggested that RH might be an age-related disease. Although the pathophysiological mechanisms of RH remain unclear, age-related damage in the mechanoreceptors of the rectal wall and afferent nerves of the pelvis and anorectum might play an important role.^13,28^

By comparison, we found that patients with RH were more likely to subjectively report obstructive symptoms, allied with much more frequent and severe straining, an a sensation of incompletion on attempted defecation. The colonic inertia scores were not different between 2 groups. The CSI scale used in this study comprises 3 subscales: obstructive defecation, colonic inertia, and pain.^19^ By comparing the CSI score between RH and non-RH patients, we could explore the relationship between RH and obstructive defecation, as well as that between RH and slow colonic transit symptoms. Next, we compared the frequency of FDD and prolonged CTT between RH and non-RH groups. We found that only FDD was more frequent in patients with RH. All these observations indicated that RH was associated strongly with pelvic floor dysfunction, but not associated with colon motility.

It is hypothesized that RH leads to FDD via the following mechanisms: because of failure of the development of an urge to defecate, the defecation reflex decreases, and the expulsive effort fails to raise.^29,30^ Over time, this leads to fecal retention and impaction. The pelvic floor muscle contracts to avoid incontinence. As a result, dysynergic defecation and poor expulsive effort lead to FDD and inadequate rectal emptying.

Recently, 2 large clinical studies were performed to detect an association between RH and outlet obstruction.^15,16^ Wijffels et al and Lee et al reported that RH was not associated with the incidence of obstructed defecation in constipated patients. It is worth noting that a number of patients included in their studies were characterized by internal rectal prolapse, rectal intussusceptions, or rectocele. For these patients, anatomical abnormalities may be more important. Lee's study included 107 FC patients, however, 21% (23 of 107) of all patients had history of spinal surgery, diabetes mellitus, or pelvic surgery (hysterectomy, ovarian surgery, and bladder surgery).^15^ Wijffels’ study only included constipated patients with high-grade internal rectal prolapse.^16^ We think that these patients do not fulfill the criteria for FC. Another important explanation for the different conclusions is that patients included in our study experienced more serious constipation symptoms with higher CSI scores compared with several other studies using the same symptom scoring system.^19,20^ The pathogenesis of refractory constipation may be more complex and RH may participate in it.

RH has been reported to be a negative predictor of the effect of BTF^14^ and surgery^31^ for fecal incontinence and constipation. However, the number of subjects in these studies was relatively small. Our data suggested that the proportion of “no effect” was significant higher in the RH group than in the non-RH group after BFT. However, the RH patients in our study had more severe obstructive symptoms and total constipation symptom severity than non-RH patients before BFT, and therefore, we could not draw the conclusion that RH influences the effect of BFT. Recently, 1 study that included 590 constipated patients was carried out to detect the association between RH and BFT efficiency.^32^ They believed that the success rate of BFT was not significantly different between RH and non-RH patients. However, they found that among the RH group, individuals who responded to BFT showed improvement of rectal sensation; among those who did not respond to BFT, rectal sensation was not improved. This change suggested that BFT restored anorectal muscle motility and rectal sensation simultaneously. These results suggested that improvement of rectal sensation leads to improvement of anorectal motility.

This is the largest cross-sectional study to detect the role of RH in constipation. We included only FC patients and excluded those with any organic diseases. We found that RH is associated with obstructive defecation symptoms and the occurrence of FDD. There were some limitations to the study. Initially, balloon distension was used to assess RH. Unfortunately, balloon distension does not always reflect true rectal afferent sensory function, as it does not consider the other important variables: rectal compliance and relaxation. Controlling the balloon insufflation using a barostat, which is an electro-mechanical device delivering isobaric rectal distention using a highly compliant polyethylene balloon, can overcome this shortcoming.^16,33^ However, the application of a barostat is limited by its relative complexity and logistics. Second, because the RH patients in our study had more severe obstructive symptoms and total constipation symptom severity than non-RH patients before BFT, we could not determine the true relationship between RH and BFT.

## CONCLUSIONS

In conclusion, RH is associated with obstructive defecation symptoms and the occurrence of FDD, but it is not associated with slow transit symptoms and delayed colon transit. Further, clinical studies are needed to detect the relationship between RH's effect on BFT and FC.
